# Development and Validation of a CD8+ T Cell Infiltration-Related Signature for Melanoma Patients

**DOI:** 10.3389/fimmu.2021.659444

**Published:** 2021-05-10

**Authors:** Yuan Yuan, Zheng Zhu, Ying Lan, Saili Duan, Ziqing Zhu, Xi Zhang, Guoyin Li, Hui Qu, Yanhui Feng, Hui Cai, Zewen Song

**Affiliations:** ^1^ School of Engineering and Applied Sciences, Harvard University, Cambridge, MA, United States; ^2^ Wenzhou Institute, University of Chinese Academy of Sciences, Wenzhou, China; ^3^ Department of Medicine, Brigham and Women’s Hospital, Boston, MA, United States; ^4^ School of Nursing, Yueyang Vocational and Technical College, Yueyang, China; ^5^ Department of Oncology, The Third Xiangya Hospital of Central South University, Changsha, China; ^6^ Xiangya School of Medicine of Central South University, Changsha, China; ^7^ College of Life Science and Agronomy, Zhoukou Normal University, Zhoukou, China; ^8^ Department of Plastic Surgery, Shanxi Bethune Hospital, Shanxi Academy of Medical Sciences, Taiyuan, China; ^9^ Department of Orthopaedics, Loudi Central Hospital, Loudi, China

**Keywords:** CD8+ T cells, melanoma, single cell RNA sequencing analysis, immunotherapy, immune response

## Abstract

**Aim:**

Immunotherapy shows efficacy in only a subset of melanoma patients. Here, we intended to construct a risk score model to predict melanoma patients’ sensitivity to immunotherapy.

**Methods:**

Integration analyses were performed on melanoma patients from high-dimensional public datasets. The CD8+ T cell infiltration related genes (TIRGs) were selected *via* TIMER and CIBERSORT algorithm. LASSO Cox regression was performed to screen for the crucial TIRGs. Single sample gene set enrichment analysis (ssGSEA) and ESTIMATE algorithm were used to evaluate the immune activity. The prognostic value of the risk score was determined by univariate and multivariate Cox regression analysis.

**Results:**

184 candidate TIRGs were identified in melanoma patients. Based on the candidate TIRGs, melanoma patients were classified into three clusters which were characterized by different immune activity. Six signature genes were further screened out of 184 TIRGs and a representative risk score for patient survival was constructed based on these six signature genes. The risk score served as an indicator for the level of CD8+ T cell infiltration and acted as an independent prognostic factor for the survival of melanoma patients. By using the risk score, we achieved a good predicting result for the response of cancer patients to immunotherapy. Moreover, pan-cancer analysis revealed the risk score could be used in a wide range of non-hematologic tumors.

**Conclusions:**

Our results showed the potential of using signature gene-based risk score as an indicator to predict melanoma patients’ sensitivity to immunotherapy.

## Introduction

Melanoma, one of the most aggressive cancers, causes approximately 61,000 deaths worldwide annually, accounting for more than 80% of skin cancer-related deaths (1). Numerous studies have revealed that the melanoma tumors are characterized by high immunogenicity and typically infiltrated by different types of immune cells (2, 3), so the melanoma tumor microenvironment (TME) consists of heterogeneous cell populations. CD8+ T cells play crucial roles in tumor suppression and elimination (4). Thus, melanoma patients with high level of immune cell infiltration, particularly CD8+ T cells, are typically shown to have favorable therapeutic outcomes and prognosis (5–8). A positive correlation between CD8+ T cell infiltration and improved prognosis is also observed in many other types of cancer (9–11), further highlighting a pivotal role of CD8+ T cells in tumor suppression. Due to the big power of natural immune system, cancer immunotherapy has emerged as a promising modality for the treatment of melanoma, with responders experiencing prolonged remission that can last several years (12, 13). One strategy of immunotherapy, known as immune checkpoint blockade, has been developed to inhibit the molecular interplay between tumor cells and immune cells. Immunotherapy with several checkpoint blockers targeting programmed cell death protein 1 (PD-1), PD-1 ligand (PD-L1), or cytotoxic T lymphocyte antigen-4 (CTLA-4) has resulted in significant improvement in clinical outcomes of melanoma patients (14). More recently, another strategy of immunotherapy called CAR-T cell therapy also shows promising effects in melanoma patients (15). Cancer vaccine, generally containing tumor-specific antigens (TSA) or tumor associated antigens (TAA), serves as another approach to generate or amplify antitumor immunity (16, 17).

However, the efficacy of these immunotherapies is restricted to only a subset of patients who have high level of CD8+ T cell infiltration (18–20), and strategies enhancing tumor-specific CD8+ T cell abundance show improved tumor suppression (21, 22). Though effective immunotherapies require infusion of large numbers of T cells (20), a comprehensive knowledge of factors affecting infiltration of CD8+ T cells into tumors, particularly melanoma, is still lacking.

With the progress of high-dimensional datasets and improvement of bioinformatics algorithm (23, 24), large-scale interrogation of gene expression and immune activity in multiple tumor types is now accessible, allowing us to investigate factors that affect CD8+ T cell infiltration and determine their correlation with patients’ survival probability as well as the response to immunotherapy.

In this work, we first screened out a list of 184 candidate genes in melanoma tumor microenvironment that were highly related to CD8+ T cell infiltration in melanoma patients. From the candidate gene list, we further identified six signature genes and constructed a signature gene-based model to generate a predictive risk score, which was strongly correlated with CD8+ T cell infiltration and validated as an independent prognostic factor of melanoma patients. Moreover, we showed the CD8+ T cell infiltration related risk score was predictive for the efficacy of immunotherapy on cancer patients. In addition to melanoma, pan-cancer analysis of 30 non-hematologic tumors further indicated that the risk score could be extensively used in other tumors. Taken together, this work might be of help in illustrating factors facilitating infiltration of CD8+ T cell into tumors and in predicting patients’ sensitivity to immunotherapy.

## Material and Methods

### Data Acquisition and Processing

Survival information (n = 463), phenotype data (n = 477) and gene expression data (HTSeq – FPKM, n = 472) of skin melanoma patients from the Cancer Genome Atlas (TCGA_SKCM) database were downloaded from the GDC hub of UCSC xena website (http://xena.ucsc.edu/public) on August 3, 2020. The first sample was selected according to the label if the same patient had two or more samples in this dataset. Normalized gene expression values were converted to transcripts per million (TPM) and log-transformed (log2(TPM+1)). Ensemble IDs were converted to gene symbols *via* the *org.Hs.eg.db* and *clusterProfiler* packages in R software. After data filtering was conducted, 448 tumor samples with survival data in the TCGA_SKCM data set were used for further analysis.

Normalized gene expression data of four melanoma-related datasets from the Gene Expression Omnibus (GEO) database (GSE65904, GSE19234, GSE22153, GSE35640 and GSE72056) were acquired *via* the *GEOquery* package in R software (25–28). For GSE65904, GSE19234, GSE22153, and GSE35640, *normalizeBetweenArrays* function of the *limma* package in R software was applied for signal intensity normalization across arrays. For a gene with multiple probes, the probe detected with the highest mean value was retained. In addition, a probe was discarded if it was mapping to two or more gene symbols. After data processing, 210 tumor samples in GSE65904, 44 samples in GSE19234, 54 samples in GSE22153 and 56 samples in GSE35640 were used for further analysis. For the single-cell RNA sequencing dataset (GSE72056), processed data file (melanoma_single_cell_revised_v2.txt) was downloaded and analyzed directly in the current study (27).

For pan-cancer analysis, normalized gene expression data (n = 10 535, TOIL RSEM tpm, unit: log2 (tpm+0.001)) and curated clinical data (n = 12 591) of the TCGA Pan-Cancer (PANCAN) cohort were downloaded from the UCSC xena website. For the evaluation of CD8+ T cell infiltration in 30 non-hematologic tumors (excluding acute myeloid leukemia (TCGA_LAML), lymphoid neoplasm diffuse large B-cell lymphoma (TCGA_DLBC), and thymoma (TCGA_THYM)), normalized gene expression data (HTSeq – FPKM) of each type of tumor was also downloaded from the GDC hub of UCSC xena website and converted to TPM.

The maf file of the simple nucleotide variation data (workflow type: MuTect2 Variant Aggregation and Masking) of the TCGA_SKCM cohort was downloaded from the GDC database (https://portal.gdc.cancer.gov/) and processed by the *maftools* package in R.

The gene expression data and clinical information of the IMvigor210 cohort was downloaded from the deposited website (http://research-pub.gene.com/IMvigor210CoreBiologies/#downloading-the-imvigor210corebiologies-package) and processed according to the instruction from the website.

### Immune Profile Analysis

The infiltration level of CD8+ T cells in each tumor sample of the 30 non-hematologic tumors from the TCGA database was evaluated by uploading TPM-normalized without log-transformation gene expression matrix into the TIMER2.0 website (http://timer.cistrome.org/), which provides robust estimation of immune infiltration levels for TCGA using six state-of-the-art algorithms, including CIBERSORT, QUANTISEQ, and xCell (23, 29). The immune and stromal scores of each sample were estimated using the ESTIMATE algorithm in the *estimate* package in R software (30). Single sample gene set enrichment analysis (ssGSEA) was conducted to evaluate the relative infiltration of 28 immune cell types in the tumor microenvironment, by using the *GSVA* package in R software (31, 32) and the feature gene panels for each immune cell type from a recent study (33). The infiltration of CD8+ T cells in each tumor sample of the GSE65094 dataset was further evaluated by uploading normalized gene expression data into the EPIC website (https://gfellerlab.shinyapps.io/EPIC_1-1/), according to the instructions from the website (34).

### Functional Analysis and Enrichment Analysis

Kyoto Encyclopedia of Genes and Genomes (KEGG) enrichment analysis was conducted using the ClueGo plug-in in the Cytoscape software (version 3.8.0). Gene set enrichment analysis (GSEA) was conducted to investigate pathways enriched in the high- and low-risk subgroups. *C2.cp.kegg.v7.1.symbols.gmt* was chosen as the gene set database. The *org.Hs.eg.db*, *clusterProfiler* and *enrichplot* packages in R software were used for analysis and visualization. The pathways were considered significantly enriched with the following criteria: nominal p-value < 0.05, q-value < 0.25, and normalized enrichment score > 1.

### Single-Cell RNA Sequencing (scRNA-seq) Analysis

Two independent scRNA-seq datasets (GSE72056 and GSE115978) were used to investigate the distribution of interested genes among different types of cells. For the GSE72056 dataset, the tumor cells and rest 7 types of non-malignant cells have been designated by the researchers of the study (27). The *Rtsne* and *ggplot2* packages in R software were used to analyze and visualize the distribution of all these types of cells. In addition, the distribution of interested genes among different types of cells in both GSE72056 and GSE115978 datasets was analyzed in the Tumor Immune Single-cell Hub (TISCH) website (http://tisch.comp-genomics.org/).

### Identification of Clusters of Melanoma Patients

The identification of clusters of melanoma patients in the TCGA_SKCM dataset was achieved by using the normalized expression of CD8+ T cells related genes and the *ConsensusClusterPlus* package in R software. Before performing consensus clustering, a filtering procedure was conducted by excluding candidate genes of low median absolute deviation (MAD) value (MAD ≤ 0.5). The specific parameters were *pam* method and sampling proportion of 0.8. The consistent matrix (CM) plots were generated for each k-value from 1 to 10. And empirical cumulative distribution function (CDF) plots revealed the consensus distributions for each k-value. The k-value at approximate maximum distribution indicated maximum stability cluster structure. The principal component analysis (PCA) was used to analyze the difference among different clusters of melanoma patients. The analysis was achieved by using the *FactoMineR* and *factoextra* packages in R software.

### Construction of the Prognostic Model

The CD8+ T cell infiltration related genes were input into the least absolute shrinkage and selection operator (LASSO) Cox regression model, and crucial gene signatures were generated *via* the *glmnet* package in R. The corresponding coefficients of the generated crucial genes were obtained through multivariate Cox analysis. The score was calculated as: score= -0.07057 * CLEC4E - 0.06700 * KLRD1 - 0.13834 * PSME1 - 0.09894 * KIR2DL4 - 0.04245 * CD274 - 0.08261 * GBP4. To facilitate the interpretation of results across data sets, the risk score was calculated with the following formula: risk score= (score-Min)/absolute (Max).

### Statistical Analysis

Correlation analysis was conducted by R software with spearman method. The median value of the risk score was used as cut-off value in dividing patients into the low- and high-risk subgroups. Univariate Cox regression and subsequent multivariate Cox regression were conducted to determine independent prognostic factors in the TCGA_SKCM dataset, by using the *survminer* package in R. The prognostic factors were further used to generate a nomogram model. The C-index of the nomogram model was calculated by the *survcomp* package in R software. The survival analyses were compared using the Kaplan–Meier method with the log-rank test. Time-dependent receiver operator characteristic (ROC) analyses and subsequent calculation of the area under the curve (AUC) were performed using the *timeROC* package in R. Wilcoxon test was conducted to compare gene expression between groups. Other packages in R used for data analysis and graph plotting included ggplot2, ggpubr, limma, vennDiagram, tidyverse, rms, dplyr and plyr. P < 0.05 was considered statistically significant (*, P < 0.05; **, P < 0.01; ***, P < 0.001; ****, P < 0.0001).

## Results

### Candidate CD8+ T Cell Infiltration-Related Genes Are Identified in Cutaneous Melanoma

**Figure 1:**
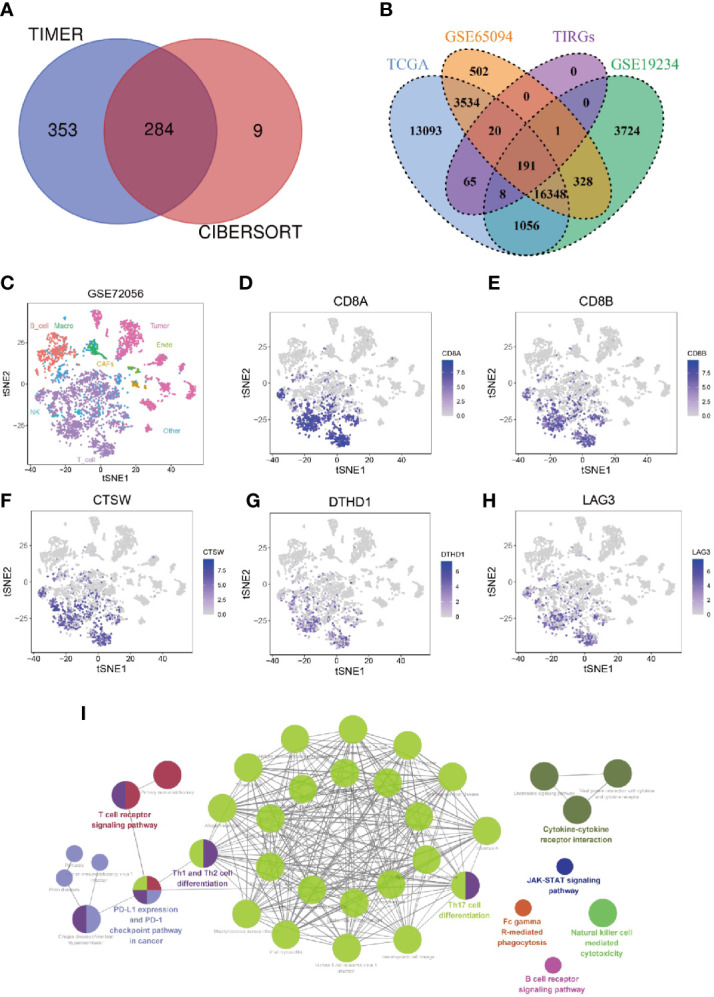
Identification and enrichment analysis of TIRGs in melanoma cells. **(A)** Venn diagram to identify TIRGs in melanoma patients from the TCGA dataset by using TIMER and CYBERSORT. **(B)** Venn diagram to screen TIRGs in melanoma patients from datasets TGCA, GSE65094 and GSE19234. **(C)** t-SNE analysis of single cell sequencing dataset GSE72056 illustrates gene expression patterns in different cell types (shown in different colors). **(D, E)** The expression pattern of CD8A **(D)** and CD8B **(E)** indicates the distribution of CD8+ T cells. **(F–H)** Illustration of three excluded TIRGs that were highly expressed in more than 50% of CD8+ T cells but less than 10% of the remaining cells in TME: CTSW **(F)**, DTHD1 **(G)** and LAG3 **(H)**. **(I)** KEGG enrichment analysis of the candidate TIRGs in melanoma TME.

Previous studies yielded inconsistent results in regarding the prognostic value of tumor infiltrating lymphocytes (TILs) in cutaneous melanoma patients (35, 36), suggesting not all types of TILs made contribution to the prognosis of these patients. To investigate the prognostic relevance of various types of immune cells in melanoma patients, we evaluated the infiltration of immune cells in cutaneous melanoma of 448 patients from TCGA_SKCM data set by TIMER and CIBERSORT, and analyzed their prognostic significance by univariate Cox analysis. As shown in **Supplementary Table 1**, the infiltration of CD8+ T cells was consistently related to patients’ survival when it was evaluated by both algorithms. In addition, high infiltration of CD8+ T cells, when estimated by four other algorithms, namely MCPCOUNTER, XCELL, QUANTISEQ and EPIC, was associated with significantly longer overall survival (**Supplementary Figures 1A–D**). We then analyzed correlation between the level of CD8+ T cell and transcriptional level of all genes in the data set. In total, we screened out 637 and 293 CD8 T cell infiltration-related genes (TIRGs) with the cutoff criteria of correlation r > 0.5 and p < 0.05 by algorithm TIMER and CIBERSORT, respectively (**Supplementary Tables 2, 3**). We selected the shared 284 TIRGs between TIMER and CIBERSORT to avoid potential bias generated by the adopted algorithms (**Figure 1A**).

To facilitate comparison in other datasets, 191 out of 284 TIRGs with expression data were selected from another two datasets GSE65094 and GSE19234 for following analyses (**Figure 1B**). Next, we analyzed the expression patterns of these 191 TIRGs in different cell types by manipulating data from the scRNA-seq dataset GSE72056. The t-SNE plot showed that there was a distinct separation among different cell clusters (**Figure 1C**), and the expression pattern of CD8A (**Figures 1D**) and CD8B (**Figures 1E**) indicated the distribution of CD8+ T cells. As the aim of this work was to understand how melanoma TME affects the infiltration of CD8+ T cells, we excluded CD8+ T cell specific markers (CD8A, CD8B, GZMK and NKG7), as well as genes expressed in most CD8+ T cells (more than 50%) but in less than 10% of the remaining cells in the melanoma TME. As a demonstration, we removed genes CTSW (**Figure 1F**), DTHD1 (**Figure 1G**) and LAG3 (**Figure 1H**), which were shown to be mainly expressed in CD8+ T cells. After removal, 184 genes were screened out as candidate TIRGs in melanoma TME. Further KEGG enrichment analysis on these 184 candidate TIRGs revealed they were mostly involved in signaling pathways such as Th17 cell differentiaction, Th1/2 cell differentiation and T cell receptor pathway (**Figure 1I**).

### Candidate TIRGs-Based Classification of Melanoma Patients

**Figure 2:**
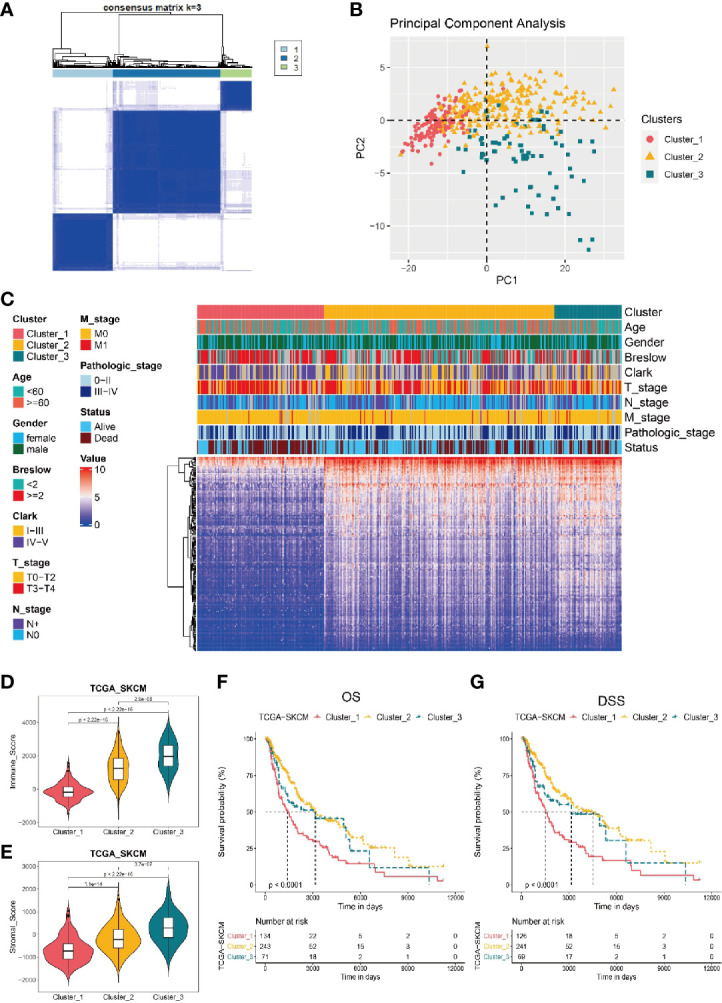
Stratificaton of melanoma patients *via* the expression of 184 TIRGs. **(A)** 448 melanoma patients from TCGA dataset are classified into three clusters based on the selected TIRGs by optima selection of unsupervised clustering. **(B)** Principle component analysis on the expression level of 184 TIRGs. **(C)** Clinical characteristics and RNA expression level of 184 TIRGs of melanoma patients from cluster 1, 2 and 3. **(D, E)** ESTIMATE analysis of immune score **(D)** and stromal score **(E)** shows a significant difference among three clusters in melanoma patients. **(F, G)** Melanoma patients in cluster 2 and 3 have significantly longer overall survival **(F)** and longer disease-specific survival **(G)** than those in cluster 1.

Based on the expression level of candidate TIRGs, we performed a consensus clustering analysis of melanoma patients from the TCGA_SKCM dataset, and k = 3 was identified from the range between 2 and 9 with optimal clustering stability (**Figure 2A**, **Supplementary Figures 1E–F**). Thus, these 448 melanoma patients from TCGA_SKCM dataset were classified into three clusters (**Supplementary Table 4**), namely cluster 1 (n = 134), cluster 2 (n = 243) and cluster 3 (n = 71). Principle component analysis (PCA) on the expression level of the 184 TIRGs further confirmed the distinction of molecular phenotype among three classified clusters (**Figure 2B**). Then we analyzed and compared the clinical characteristics, gene expression level of the 184 TIRGs (**Figure 2C**) and immune cell infiltration level (**Supplementary Figure 1G**) among these three clusters. The results showed that melanoma patients from patients from cluster 1 had significantly different clinical characteristics and immune cell infiltration level than those from either cluster 2 or cluster 3.

To confirm the difference of immune activity among these three clusters, we compared the immune score (**Figure 2D**) and stromal score (**Figure 2E**) by ESTIMATE, which are used to represent the infiltration of immune cells and the presence of stroma in melanoma. Consistent with previous analysis results, melanoma patients from cluster 1 to 3 showed an increasing immune score and stromal score, indicating an increasing immune activity. Furthermore, we analyzed the survival rate of melanoma patients from these three clusters, and the results revealed that melanoma patients in cluster 2 and 3 had significantly longer overall survival (**Figure 2F**) and longer disease-specific survival (**Figure 2G**) than those in cluster 1. Similar results were obtained in dataset GSE65904 (**Supplementary Figures 2A–F**).

Taken together, these data indicated that three clusters of melanoma patients classified by the 184 candidate TIRGs had distinct immune cell infiltration level, immune activity and survival probability. Based on the properties of immune activity and survival probability, melanoma patients from cluster 2 and 3 were combined together as the ‘hot-tumor’ melanoma group, while those from cluster 1 was referred as ‘cold-tumor’ melanoma population in the following analysis.

### A Representative Risk Score for Patient Survival Is Constructed Based on Six Signature Genes

**Figure 3:**
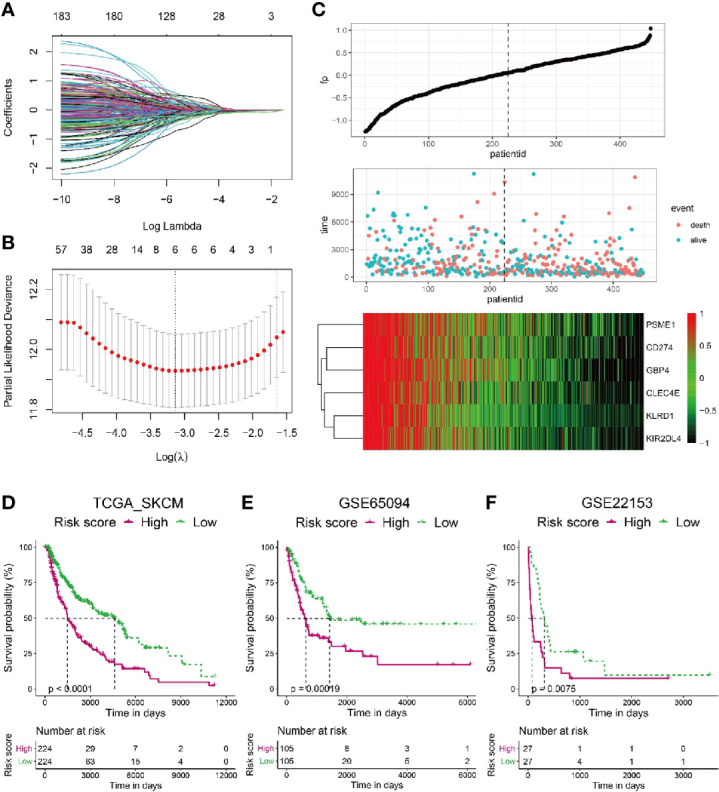
Construction of a risk score based on six signature genes. **(A, B)** The LASSO Cox regression model was constructed from 184 signature genes, and the tuning parameter (λ) was calculated based on the partial likelihood deviance with ten-fold cross validation. The six signature genes were identified according to the best fit profile. **(C)** Melanoma patients in the TCGA training set were divided into two populations according to the median value of the risk scores. **(D–F)** Melanoma patients with lower risk score have significantly longer survival in the TCGA whole set **(D)**, GSE65094 data sets **(E)** and GSE22153 data sets **(F)**.

To construct a more applicable classifier in reflecting distinct infiltration level of CD8+ T cells, immune status and prognosis of melanoma patients, we first analyzed the prognostic significance of all these 184 TIRGs by univariate Cox analysis, which suggested that these genes were all protective factors (**Supplementary Table 5**). Then, we performed LASSO Cox regression analysis of these 184 TIRGs in the whole TCGA_SKCM dataset. As a result, six signature genes, *CLEC4E, PMSE1, CD274, KLRD1, KIR2DL4 and GBP4*, were generated based on the optimal value of λ (**Figures 3A, B**). We further analyzed their expression in different cell types (**Supplementary Figure 3**). Gene *PMSE1* has a wide expression in most immune cells, endothelial cells, fibroblasts, and malignant cells, while *KLRD1* and *KIR2DL4* are shown to be mainly expressed in CD8ex cells. Some of the CD8Tex and CD4Tconv cells are shown to have expression of gene *GBP4* and *CD274*, while *CLEC4E* is mainly expressed in Mono/Macro cells. It is worth to note that all these genes except *GBP4*, are reported to have a direct or indirect influence on immune cell infiltration, and some of them have been used as prognostic marker in different cancers (37–41).

To integrate information of these six signature genes, a novel score was calculated by multiplying their expression level with the corresponding coefficients by multivariate Cox analysis. The risk scores were then obtained by the formula mentioned in the Materials & Methods section. To investigate if the risk score was predictive for patients survival, we splitted the melanoma patients in the whole TCGA_SKCM dataset into two populations with a cutoff of risk score at the median level. The patients with risk score higher than the median level were included in high risk population, while the patients with risk score lower than the median level fell into low risk population. As shown in **Figure 3C**, patients with high risk score tended to have more occurrence of death and low expression of all the six signature genes.

Kaplan–Meier analysis revealed that melanoma patients from the low risk population had sinigificantly longer OS than those from the high risk one in the TCGA_SKCM dataset (**Figure 3D**, p < 0.0001). Moreover, we calculated the risk scores for melanoma patients from another two datasets as additional validations, GSE65094 (**Figure 3E**) and GSE22153 (**Figure 3F**), respectively. Consistently, patients with low risk score had higher survival probability than those with high risk score, confirming the patient survival probability could be reflected by the risk score based on the selected six signature genes.

We further compared the predictiblibity of this signature with other previously developed immune-related signatures. Eleven studies were screened out after literature searching (42–52), but five signatures were not included for further analyses for at least one of the following reasons: lack of formula to calculate the risk score or immune-related score; lack of validation in extra datasets; lack of RNA expression data of some signature genes in validated datasets in the current work (45–48, 52). As reflected by the AUC values at 1-year, 3-year and 5-years in **Supplementary Figures 4A–D** and **5A–C**, the signatures developed in our work and Tian’s work had relatively better performance in predicting outcome of melanoma patients.

### The Risk Score Serves as an Indicator for CD8+ T Cell Infiltration in Melanoma Patients

**Figure 4:**
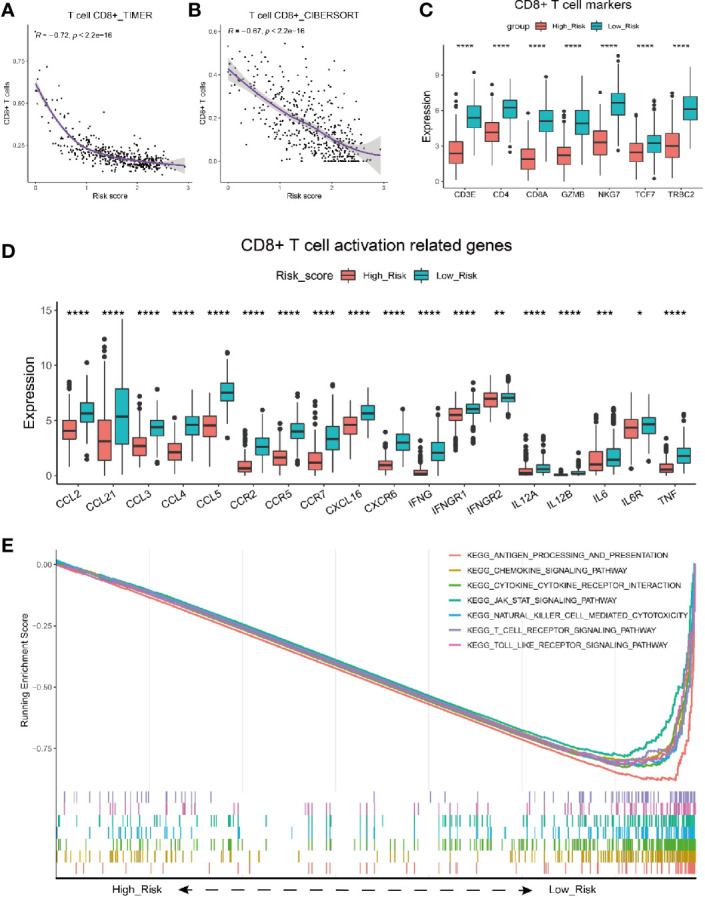
Characterization of risk score with immune activity. **(A, B)** Correlation between risk score and the infiltrating number of CD8 T cells in melanoma patients by analysis with TIMER **(A)** and CIBERSORT **(B)**. **(C)** Comparison of seven T cell marker expression level between melanoma patients with high and low risk scores in the TCGA cohort. **(D)** Relationship between risk score and CD8+ T cell activation related genes. Adjusted P values were showed as: *P < 0.05; **P < 0.01; ***P < 0.001, ****P < 0.0001. **(E)** GSEA of gene expression from data sets TCGA_SKCM shows genes involving in CD8+ T cell infiltration signaling pathway mostly enriched in melanoma patients with low risk scores.

Next, we adopted different algorithms to evaluate the infiltration of CD8+ T cell and analyzed its correlation with the risk score. The results consistently showed that there was a strong negative correlation between the risk score and CD8+ T cell infiltration (**Figures 4A, B**, **Supplementary Figures 6A–D**). Moreover, we analyzed the expression level of gene *CD3E, CD4, CD8A, GZMB, NKG7, TCF7* and *TRBC2*, the well-known markers for CD8+ T cells, and they were all shown to have significantly higher expression level in melanoma patients with low risk (**Figure 4C**). To expand on these data, we further investigated whether particular T cell functions (cytokines/chemokines, for example) were differentially correlated with the risk score. We analyzed the correlation between the risk score and a bunch of cytokines/chemokines which are positively related with T cell infiltration functions (53). Intriguingly, almost all of these functional cytokines/chemokines showed significantly higher expression level in patients with low risk scores (**Figure 4D**), indicating the signature could also predict the activity or functional status of T cells within the tumor. As the representative index for immune activity, immune score and stromal score was analyzed for melanoma patients with low and high risk score. The results showed that melanoma patients with low risk score had a significantly higher immune score (**Supplementary Figure 6E**) and stromal score (**Supplementary Figure 6F**) than those with high risk score.

To further validate the correlation between risk score and CD8+ T cell infiltration, we performed analysis on melanoma patients from GSE65094. Similarly, the risk score had a strongly negative correlation with the infiltrating CD8+ T cells (**Supplementary Figure 6G**, R=-0.67). The expresson level of CD8+ T cells markers including CD8A, GZMB, NKG7, etc., was all shown to be higher in low risk melanoma patients (**Supplementary Figure 6H**). Besides, Melanoma patients with low risk scores had significantly higher immune scores (**Supplementary Figure 6I**) than those with high risk score.

Furthermore, we performed GSEA analysis of gene expression data from TCGA_SKCM dataset (**Figure 4E**). Consistently, The results showed that the genes mainly enriched in low risk population were typically involved in T cell activation/infiltration process, including the antigen processing and presentation, chemokine signaling pathway, cytokine-cytokine receptor interaction, T cell receptor signaling pathway, etc. Taken together, the risk score was validated as a good indicator for immune activity especially CD8+ T cell infiltration in melanoma patients.

### Analysis of Tumor Mutation Burden Is Performed for High and Low Risk Group

**Figure 5:**
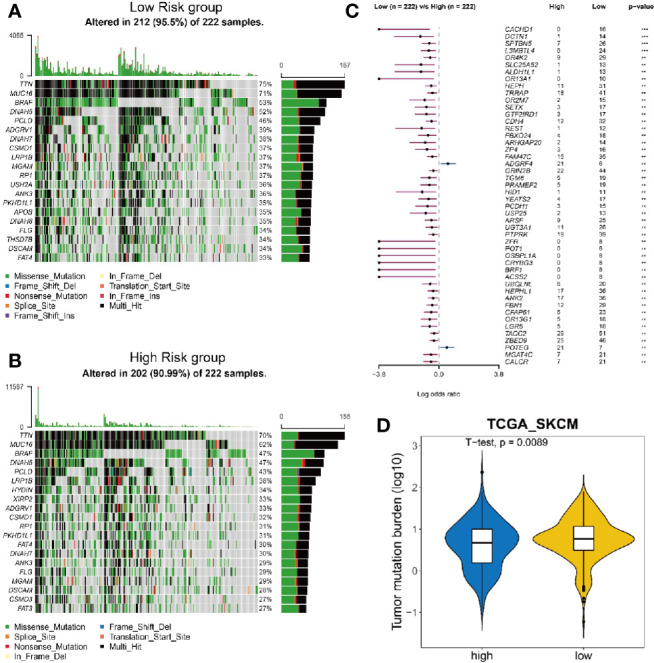
Analysis of mutation burden in high and low risk group. **(A, B)** Mutation landscape of 222 melanoma tumor samples with low risk **(A)** and high risk **(B)**. Central matrix shows somatic mutations with colors indicating different types of mutations and genes mutated at high frequency are represented in the left list. The top bar plot shows the number of gene mutations in each sample and the mutation rate of significantly mutated genes is displayed on the right. **(C)** Comparison of mutation frequency between high risk group and low risk group. **(D)** Comparison of tumor mutation burden between high risk group and low risk group.

Since highly mutated tumors can produce many antigens, which would stimulate T cells to respond to the antigens and mount an anti-tumor response, we wondered whether the risk score could also reflect the tumor mutation burden (TMB). We analyzed the mutation frequency of all genes from low (**Figure 5A**) and high (**Figure 5B**) risk group of 222 melanoma tumor sampls. The results show that the mutation frequency is significantly higher in low risk group than that in high risk group (**Figure 5C**). Consequently, the low risk group had a significantly higher TMB (**Figure 5D**), suggesting that the risk score can also reflect the level of mutation burden.

### Risk Score Acts as an Independent Prognostic Value for the Survival of Melanoma Patients

The preceding analyses suggested a tight correlation between the risk score and CD8+ T cell infiltration; this spurred an interest to analyze the associations between the risk score and clinicopathological features of melanoma patients. As shown in **Supplementary Table 6**, more patients in the high risk population were male (p = 0.0403), with a Breslow depth larger than 2 cm (p < 0.0001), at an advanced Clark level (IV or V, p = 0.0006), at an advanced T stage (T3-T4, p < 0.0001), and with a dead status (p < 0.0001).

**Figure 6:**
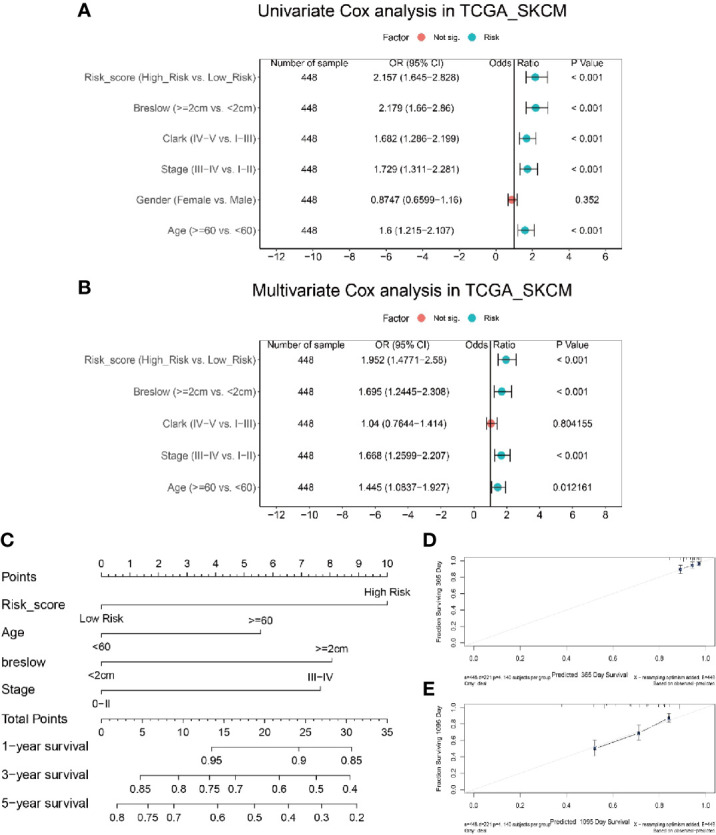
Integration analysis of risk score and clinical characteristics to predict the survival of melanoma patients. **(A)** Univariate analysis shows only gender is not significantly correlated with disease progression. **(B)** Multivariate analysis shows risk score, breslow, and age is significantly correlated with disease progression. **(C)** Nomogram including risk score constructed to predict the 1-, 3-, and 5-year survival of melanoma patients in the TCGA cohort. **(D, E)** Calibration curve of the nomogram for predicting the probability of OS at 1 and 3 years.

To determine whether the risk score was an independent prognostic predictor for OS, we performed univariate and multivariate Cox regression analysis among the available variables. In univariate Cox regression analyses, the risk score was significantly associated with OS in the TCGA_SKCM cohort (OR = 2.157, 95% CI = 1.645-2.828, P < 0.001) (**Figure 6A**). After correction for other confounding factors, the risk score was proved to be an independent predictor for OS in the multivariate Cox regression analysis (TCGA_SKCM cohort: OR =1.952, 95% CI = 1.477-2.58, P < 0.001) (**Figure 6B**). Breslow depth, stage and age were also shown to be independent predictiors for OS (**Figure 6B**).

In order to establish a quantitative approach to predict the survival of melanoma patients, we integrated the risk score and other independent clinical risk factors (Breslow depth, age, and stage) to construct a nomogram (**Figure 6C**). A point scale of the nomogram was utilized to dispense points to respective variables based on multivariate Cox analysis. We drew a horizontal straight line to determine the points for each variable, and the total points of each patient were calculated by adding the points of all variables together, which were normalized to a range from 0 to 35. The estimated survival rates at 1, 3, and 5 years of melanoma patients were calculated by drawing a vertical line between the total point related axis and each prognostic related axis. The results of the calibration plots indicated that there was good consistency between the predicted and the actually observed outcomes (**Figures 6D, E**). The C-index of the nomograph was calculated to be 0.70 (0.66 – 0.74), suggesting a good predictability. The predictive performance of this nomogram was also compared with that of individual risk factors, and the results indicated that the nomogram performance was better than that of risk score (C-index: 0.69), age (C-index: 0.59), breslow depth (C-index: 0.69) and stage (C-index: 0.67) alone. Consequently, our results suggested that the nomogram was an optimal model to predict the survival of melanoma patients.

### Risk Score Is Shown to be Predictive for the Efficacy of Immunotherapy on Melanoma Patients

**Figure 7:**
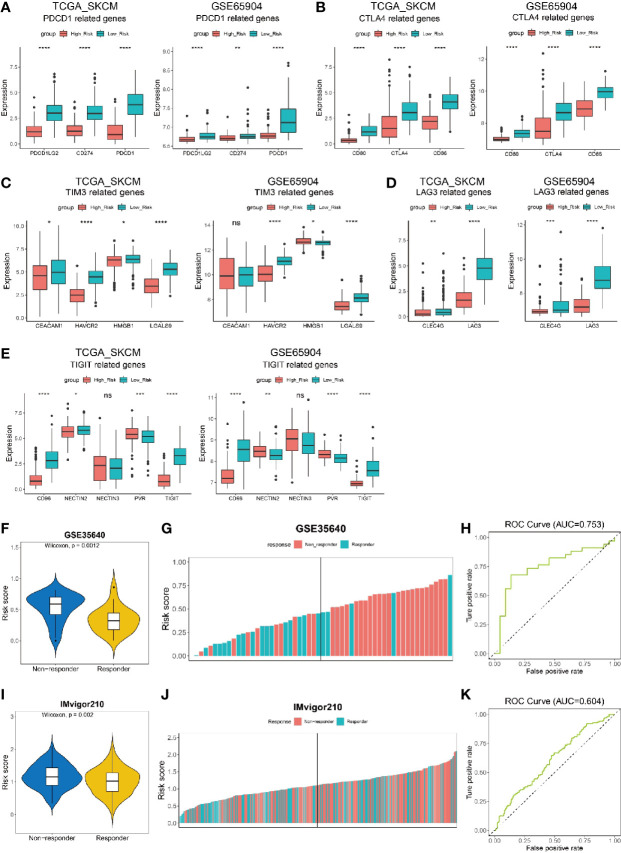
Risk score to predict the efficacy of immunotherapy on cancer patients. **(A–E)** Expression of immunotherapy targeted-genes in low risk and high risk melanoma patients from datasets TCGA_SKCM and GSE65904. **(A)** PDCD1-related genes. **(B)** CTLA4-related genes. **(C)** TIM3-related genes. **(D)** LAG3-related genes. **(E)** TIGIT-related genes. **(F, G)** Melanoma patients from data set GSE35640 who received immunotherapy but have no response (non-responder) show higher risk scores than those responders. **(K)** ROC curve showing the performance of our model for predicting the efficacy of immunotherapy on urothelial cancer patients in data set IMvigor210 at all classification thresholds (AUC=0.604) at all classification thresholds (AUC=0.753). **(I, J)** Non-responding urothelial cancer patients from data set IMvigor210 show higher risk scores than those responders. **(H)** ROC curve showing the performance of our model for predicting the efficacy of immunotherapy on urothelial cancer patients in data set IMvigor210at all classification thresholds (AUC=0.604). ns, not significant; *P < 0.05; **P < 0.01; ***P < 0.001; ****P < 0.0001.

As risk score could indicate the immune activity, we furthter investigated the expression level of five hot immunotherapy targeted genes in melanoma patients with low and high risk score from datasets TCGA_SKCM and GSE65904. We found PDCD1LG2, CD274 and PDCD1 genes, which are all related to PD-1/PD-L1 signaling pathway that inhibit T cell function, had significantly higher expression level in low risk population than high risk population (**Figure 7A**). Similarly, CD80, CTLA4 and CD66 genes that involve in CTLA4/CD80-86 signaling pathway for T cell inhibition were also significantly highly expressed in low risk melanoma patients (**Figure 7B**). In addition, TIM3/TIM3L (**Figure 7C**) and LAG3/LAG3L (**Figure 7D**) signaling pathway related genes were all shown to have higher expression level in low risk population. In addition, for TIGIT/CD96 signaling pathway, most of the related genes were shown to have high expression levels in low risk melanoma patients (**Figure 7E**). In summary, the correlation between risk score and the expression level of genes that related to immunotherapy targeted signaling pathway in melanoma patients indicated the potential of risk score to predict patients’ sensitivity to the corresponding immunotherapy.

To validate if the risk score was predictive for the efficacy of immunotherapy, we analyzed the risk score for melanoma patients who received MAGE-A3 cancer immunotherapeutic from dataset GSE35640. The patients were divided into two populations, responder and non-responder, representing the immunotherapy had effect or not on melanoma patients, respectively. Our results showed that the non-responder population had a significantly higher risk score than the responder either from dataset GSE35640 (**Figure 7F**). Additionally, the patients were ranked by risk score and a higher percentage of responder was found in melanoma patients with low risk score (left half shown in **Figure 7G**). Further analysis showed that the risk score was predictable for the patients’ response to immunotherapy in dataset GSE35640 (**Figure 7H**, AUC=0.753), indicating risk score had a good predictablity in evaluating the sensitivity of melanoma patient towards immunotherapy like MAGE-A3 cancer immunotherapeutic. Another dataset, the IMvigor210 cohort, contained comprehensive RNA expression data and clinical information of 297 patients with metastatic urothelial cancer who were treated with an anti-PD-L1 agent (atezolizumab) (54). We also calculated the risk score in the dataset and noticed that patients who were responders (complete response or partial response) to the immunotherapy had significantly lower risk score than those who were non-responders (stable disease or progression disease) to the treatment (**Figure 7I**, p = 0.002). More patients in the low risk group were found to be responders to atezolizumab (left half shown in **Figure 7J**), and the risk score was predictable for urothelial cancer patients’ response to the anti-PD-L1 agent in this dataset (**Figure 7K**, AUC=0.604).

### Pan-Cancer Analysis Revealed the Risk Score Can Be Used in a Wide Range of Non-Hematologic Tumors

**Figure 8:**
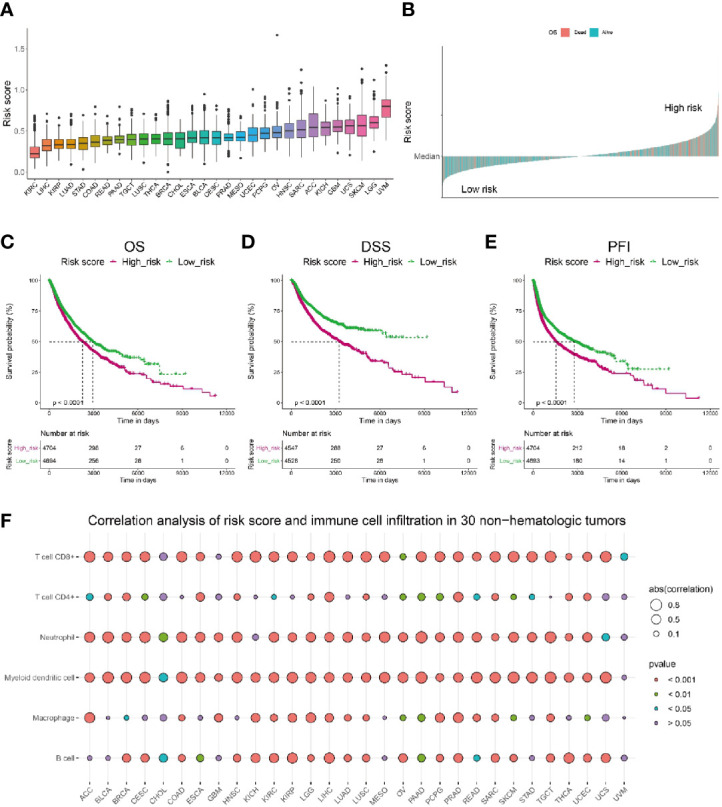
Pan-cancer analysis of the correlation between risk score and immune cell infiltration as well as patients’ survival. **(A)** Pan-cancer analysis of risk score in 30 non-hematologic tumors. **(B)** Pan-cancer analysis of risk score indicated patients with high risk (above median) had higher death rate (shown in pink). **(C–E)** Pan-cancer analysis showed that patients with lower risk score have significantly longer overall survival **(C)**, longer disease-specific survival **(D)** and longer disease-free interval **(E)** than those with higher risk score. **(F)** Correlation analysis of risk score and immune cell infiltration level in 30 non-hematologic tumors by TIMER.

To investigate if the risk score could be generalized to other tumors, we performed a pan-cancer analysis on risk score of 30 non-hematologic tumors (**Figure 8A**). Among these tumors, uveal melanoma (UVM) was shown to have the highest risk score, whereas Kidney Renal Clear Cell Carcinoma (KIRC) was ranked with the lowest risk score. Interestingly, current clinical trials of immunotherapy on UVM patients showed a very low efficacy: the overall response rate of UVM patients treated with PD-1 or PD-1 ligand (PD-L1) inhibitors was 3.6% and the median overall survival was 7.6 months (55). In contrast, patients with KIRC and LIHC, whose risk scores were ranked as the lowest two tumors in our model, got better responses to immunotherapies (56, 57). The results of current clinical trials of immunotherapies on different tumors seemed consistent with our risk score analysis, indicating the risk score might have a good performance in predicting the sensitivity to immunotherapies not only for melanoma patients, but also for other tumors.

Further pan-cancer analysis showed a higher death rate in cancer population with risk score higher than the median level, which was referred as high risk population (**Figure 8B**). Consistently, we compared the survival probability of patients from high risk and low risk population, and the results revealed that the high risk population had significantly shorter overall survival (**Figure 8C**), shorter disease-specific survival (**Figure 8D**) and shorter disease-free interval (**Figure 8E**) than those of low risk population. Moreover, we calculated the correlation between risk score and immune cell infiltration level in these 30 non-hematologic tumors and found a distinct negative correlation (r < -0.5) between risk score and CD8+ T cell infiltration in most of the tumors (20/30, **Figure 8F**, **Supplementary Table 7**). Besides CD8+ T cells, the infiltration of myeloid dendritic cells and neutrophil cells also had significant negative correlations with risk score (**Figure 8F**, **Supplementary Table 7**). Taken together, the risk score could be used extensively in other tumors besides melanoma.

## Discussion

Immunotherapeutic approaches to melanoma treatment become increasingly widespread. Despite the big advance, the efficacy of immunotherapy is not equal to every melanoma patient. Early studies found that ‘sufficient’ T cell infiltration in tumor sites is critical for the response to anti-PD-L1 therapy, and that poor representation of CD8+ T cells in tumors is a fundamental hurdle to successful immunotherapy (20, 58). Thus, T cell infiltration level can be used to indicate melanoma patients’ sensitivity to immunotherapies. Since previous studies reported inconsistent results about the prognostic value of TILs in melanoma patients, we hypothesize that not all types of TILs contribute to melanoma patients’ survival. In this work, we firstly investigated the prognostic relevance of the infiltration of various types of immune cells in melanoma patients and found that infiltration of CD8+ T cells had significant prognostic value when it was estimated by different algorithms (**Supplementary Table 1**, **Supplementary Figures 1A–D**). We then focused on identifying genes that were highly correlated with CD8+ T cell infiltration in melanoma. It should be noted that CD8+ T cell specific marker genes, as well as the genes that were expressed in most CD8+ T cells whereas in less than 10% of the rest cells, were excluded from the screened gene list, as we hope to extract genes from melanoma microenvironment that facilitate the infiltration of CD8+ T cells. As a result, 184 genes were filtered out as the candidate TIRGs (**Supplementary Tables 2**, **3**). Some of these genes have been proved to facilitate the infiltration of CD8+ T cells into tumors. For instance, active secretion of CXCL10 and CCL5 from TME is found to be associated with high infiltration of cytotoxic CD8+ T cells in colon cancer, and chemotactic migration of CD8+ T cells towards esophageal squamous cell carcinoma is greatly hampered with the treatment of anti-CXCL10 or anti-CCL5 neutralizing antibodies (59, 60). Roberto S. Accolla and colleagues revealed that immune cells including CD4+ T cells, CD8+ T cells, dendritic cells and macrophages would be rapidly infiltrated into tumor cells that were stably transfected with CIITA and had expression of MHC class II molecules (61–63). In addition, EBI3-deficient C57BL/6 mice injected with B16 melanoma cells exhibits a significantly increased tumor growth relative to wild-type control mice, and tumors from *EBI3^-/-^* mice contains significantly decreased proportions of CD8+ T cells, suggesting a positive role of EBI3 in the infiltration of these cytotoxic T cells (64).

Current knowledge of the tumor–immune system interaction has been applied for the stratification of cancer patients (65). Immunoscore — a standardized scoring system based on the level and spatial distribution of CD3+ and CD8+ T cell infiltration, has been developed to classify tumors into three major types, namely ‘cold’, ‘altered’ and ‘hot’ immune tumors (65, 66). With the development of bioinformatics methods, a more precise classification of tumor types can be achieved by comprehensive analysis of the immune landscape in tumors through bulk gene expression profiling (23, 67, 68). Consequently, the terms ‘hot’, ‘altered’ and ‘cold’ are now typically referred to T cell-infiltrated, inflamed but non-infiltrated, and non-inflamed tumors, which has been validated in melanoma (65, 69). In this work, melanoma patients from the TCGA_SKCM or GSE65904 cohort were classified into three clusters based on the expression of 184 candidate TIRGs by consensus clustering analysis (**Figure 2A**, **Supplementary Figure 2A**). Cluster 2 and cluster 3 were referred as ‘hot’ tumors due to their high immune activity and survival probability, whereas cluster 1 with low immune activity and survival probability was defined as ‘cold’ tumor.

The capability of these 184 TIRGs in stratifying melanoma patients into subgroups with distinct prognosis and immune activity prompted us to construct a more applicable prognostic classifier. Six signature genes were finally selected *via* the LASSO Cox regression analysis, and were used to construct a CD8+ T cell infiltration related risk score (**Figures 3A, B**). To validate if the risk score can truly reflect the immune activity, especially the level of CD8+ T cell infiltration, and survival probability of melanoma patients, we elaborated integration analyses on melanoma patients from several independent datasets by using different algorithms (TIMER, CIBERSORT, MCPCOUNTER, QUANTISEQ, EPIC and ESTIMATE) and Kaplan–Meier analysis. All these six genes except *GBP4*, are reported to have a direct or indirect influence on immune cell infiltration, and some of them have been used as prognostic marker in different cancers (37–41). However, the specific mechanism of how these genes influence T cell function remains elusive, it would be very interesting to further investigate into each of them in the following study. In this work, we found the combination of these six genes had a novel and good predictivity on melanoma patients, which performed better than the existing signature genes (**Supplementary Figures 4**,** 5**) (43, 44, 49–51). Although the six gene signature in our work had a similar performance with the signature from Tian’ s study for melanoma patients’ survival, our signature had a better capability in predicting patients’ response to immunotherapy (**Figures 7H, K**, **Supplementary Figures 7A–C**).

Moreover, the nomogram, a comprehensive evaluation combining the risk score with other important clinical variants (Breslow depth, stage and age), showed a favorable consistency between the actual and predicted values for 1-, 3-, and 5-year OS. The C-index of the nomogram was higher than that of the individual risk factors, suggesting the nomogram might be an optimal and valuable new prognostic method for clinicians in the future.

In order to improve the treatment efficiency, it will be critical to predict the efficacy of immunotherapy in melanoma patients. As effectiveness of immunomodulatory strategies depends on the pre-existence of anti-tumor CD8+ T cells (65), we proposed that the risk score developed in this work can predict patients’ response to immune checkpoint blockade inhibitors (ICIs) due to its strong inverse correlation with CD8+ T cell infiltration and the tumor mutation burden. In addition, melanoma patients with low risk have a significantly up-regulation of a number of inhibitory receptors and ligands (**Figures 7A–E**), which usually leads to T cell exhaustion and dysfunction (70–72). Strategies targeting these immune-inhibitory pathways were expected to rescue exhausted T cells into a cytotoxic phenotype, and enhance T cell proliferation and differentiation, leading to tumor suppression and elimination. Indeed, early studies showed that melanoma patients with higher expression of PD-L1 have a higher objective response rate (ORR) and longer survival time when treated with pembrolizumab (73). Moreover, the risk score indicated that combinational use of ICIs targeting different immune-inhibitory pathways might be more effective to melanoma patients with low risk. The anti-CTLA4–PD-1 dual immunotherapy has been successful in the treatment of a set of tumors including melanoma (65). The median OS of melanoma patients was longer than 60.0 months (median not reached) in the nivolumab-plus-ipilimumab group and 36.9 months in the nivolumab group, as compared with 19.9 months in the ipilimumab group (74). Other combination of ICIs, like anti-PD-1 plus LAG3 blockade, has yielded synergistic potential in preclinical models (75). In addition, our work showed that the risk score has a good predictability of treatment response in melanoma patients receiving MAGE-A3 cancer immunotherapeutic (**Figures 7F–H**). Although the cancer vaccine is shown inefficacious in melanoma patients in a recent phase 3, double-blind, randomised, placebo-controlled trial (76), its therapeutic effect in the low-risk subgroup of melanoma patients warrants further clicnial investigation. Even in the non-melanoma cohort, the risk score could also be used to predict patients’ response to immunotherapy (**Figures 7I–K**). Taken together, melanoma patients with low risk, characterized by high infiltration of CD8+ T cells and high expression of multiple immune inhibitory receptors and ligands, should be more sensitive to immunotherapy, either monotherapy or a combined therapy.

Moreover, pan-cancer analysis showed that the risk score constructed in melanoma has a strong inverse correlation with CD8+ T cell infiltration in many non-hematologic tumors (**Figure 8F**), indicating the risk score can be extensively used in a variety of tumors. Cancer patients with high risk have a significantly higher mortality and shorter OS, DSS and PFI in spite of tumor origins (**Figures 8B–E**).

As it was reported in 2011 that the type, density and location of immune cells within the tumor site could predict survival of patients with colorectal cancer more accurately than the classical TNM system, evaluation of the immune landscape in tumors, such as the Immunoscore, has been proved to show a greater relative prognostic value than traditional clinical features (65). On the other hand, the advent of cancer immunotherapies has revolutionized the field of oncology and benefitted more cancer patients, leading to an improved survival time. Therefore, immune-related prognostic classifier should have better predictability of treatment response and prognosis of cancer patients.

At last, it should be pointed out that the current study had some limitations. Firstly, the cause-and-effect relationship between the selected six signature genes and the infiltration of CD8+ T cells warrants further investigation. It is possible that high level of some of these genes does not facilitate infiltration of CD8+ T cells but reflects a feedback to the infiltration of the cytotoxic immune cells, as suggested by an early study (77). In addition, the current study was a retrospective analysis, thus the value of the risk score in predicting melanoma patients’ survival and response to immunotherapy should be validated in a large and perspective study.

In conclusion, we have constructed a risk score to predict patients’ response to immunotherapy, which might be used as a clinical index to pre-evaluate the efficacy of immunotherapy. We believe with the growing availability of high-dimensional database and bioinformatics approaches; the accuracy of prediction would be further improved and can have a better guidance in personalized immunotherapeutic approaches.

## Data Availability Statement

The original contributions presented in the study are included in the article/**Supplementary Material**. Further inquiries can be directed to the corresponding author.

## Ethics Statement

Ethical review and approval was not required for the study on human participants in accordance with the local legislation and institutional requirements. Written informed consent for participation was not required for this study in accordance with the national legislation and the institutional requirements.

## Author Contributions

YY and ZZ contributed equally to the study, which was conceived and designed by ZS and YY. Data analysis was carried out by YY and ZZ. Bioinformatics analysis with R software was conducted by ZS, YL, XZ, and GL. The searching and reading of the existing literature were conducted by SD, ZQZ, YL, YF, and HC. YY and ZS drafted the article. All authors contributed to the article and approved the submitted version.

## Funding

This work was supported by the Youth Program of the National Natural Science Foundation of China (Grant No. 81802940).

## Conflict of Interest

The authors declare that the research was conducted in the absence of any commercial or financial relationships that could be construed as a potential conflict of interest.
