# Supplementary material for: Development and Validation of a CD8+ T Cell Infiltration-Related Signature for Melanoma Patients
Source: Front Immunol. 2021 May 10;12:659444. doi: 10.3389/fimmu.2021.659444 (PMC8141567; doi:10.3389/fimmu.2021.659444)
Supplement: Supplementary file 2 [file DataSheet_2.docx]

**Supplementary Figure legend**

**Supplementary Figure 1. Clustering analysis of melanoma patients from dataset TCGA_SKCM.** (A-D) Melanoma patients with high infiltration of CD8+ T cells have significantly longer survival analyzed by MCPCOUNTER (A), XCELL (B), QUANTISEQ (C) and EPIC (D). (E) Consensus empirical cumulative distribution function (CDF) of all given cluster numbers from dataset TCGA_SKCM. (F) Plot of delta area changes with number of clusters from dataset TCGA_SKCM. (G) ssGSEA data shows an increasing level of immune cell infiltration in cluster 1, 2 and 3.

**Supplementary Figure 2. Clustering analysis of melanoma patients from dataset GSE65904.** (A) Consensus clustering displaying the robustness of classification from dataset GSE65904. (B) Consensus empirical cumulative distribution function (CDF) of all given cluster numbers from dataset GSE65904. (C) Plot of delta area changes with number of clusters from dataset GSE65904. (D) Survival probability of melanoma patients in three clusters from dataset GSE65904. (E-F) Immune score (E) and stromal score (F) of melanoma patienets in three clusters from dataset GSE65904.

**Supplementary Figure 3. Expression pattern analysis of six signature genes in datasets SKCM_GSE72056 and SKCM_GSE115978_aPD1.** (A and B) t-SNE analysis of dataset SKCM_GSE72056 (A) and SKCM_GSE115978_aPD1 (B) illustrates gene expression patterns in different cell types (shown in different colors). (C) Expression pattern of signature genes *PSME1*, *KLRD1, KIR2DL4,* *GBP4*, *CLEC4E* and *CD274* in datasets GSE72056. (D) Expression pattern of signature genes *PSME1*, *KLRD1, KIR2DL4,* *GBP4*, *CLEC4E* and *CD274* in datasets GSE115978.

**Supplementary Figure 4. Comparisons of the predictivity between this study and other studies.** (A) Predictivity of our risk score in melanoma patients’ 1-year, 3-year and 5-year survival from dataset TCGA_SKCM, GSE65904 and GSE22153. (B) Predictivity of Liu’s study in melanoma patients’ 1-year, 3-year and 5-year survival from dataset TCGA_SKCM, GSE65904 and GSE22153. (C) Predictivity of Hu’s study in melanoma patients’ 1-year, 3-year and 5-year survival from dataset TCGA_SKCM, GSE65904 and GSE22153. (D) Predictivity of Tian’s study in melanoma patients’ 1-year, 3-year and 5-year survival from dataset TCGA_SKCM, GSE65904 and GSE22153.

**Supplementary Figure 5. Comparisons of the predictivity between this study and other studies.** (A) Predictivity of Song’s study in melanoma patients’ 1-year, 3-year and 5-year survival from dataset TCGA_SKCM, GSE65904 and GSE22153. (B) Predictivity of Wan’s study in melanoma patients’ 1-year, 3-year and 5-year survival from dataset TCGA_SKCM, GSE65904 and GSE22153. (C) Predictivity of Yan’s study in melanoma patients’ 1-year, 3-year and 5-year survival from dataset TCGA_SKCM, GSE65904 and GSE22153.

**Supplementary Figure 6. Relation between risk score and immune cell infiltration.** (A-D) Correlation between risk score and the infiltrating number of CD8 T cells in melanoma patients from SKCM_TCGA dataset by analysis with XCELL. (A), QUANTISEQ (B), MCPCOUNTER (C) and EPIC (D). (E and F) ESTIMATE analysis of immune score (E) and stromal score (F) shows a significant difference between high risk and low risk melanoma patients. (G) Correlation between risk score and the infiltrating number of CD8 T cells in melanoma patients from GSE65094 dataset by analysis with EPIC. (H) Expression level of CD8+ T cell markers in high risk and low risk melanoma patients from GSE65094 dataset. (I) ESTIMATE analysis of immune score shows a significant difference between high risk and low risk melanoma patients from GSE65094 dataset. *, P< 0.05; **, P< 0.01; ***, P< 0.001, ****, P< 0.0001.

**Supplementary Figure 7. Tian’s signature to predict the efficacy of immunotherapy on cancer patients.** (A) ROC curve showing the performance of Tian’s signature for predicting the efficacy of immunotherapy on melanoma patients in data set GSE35640 at all classification thresholds (AUC = 0.726). (B) Non-responding urothelial cancer patients from data set IMvigor210 showed no difference in risk score, based on Tian’s signature, when compared with the responders. (H) ROC curve showing the performance of Tian’s signature for predicting the efficacy of immunotherapy on urothelial cancer patients in data set IMvigor210at all classification thresholds (AUC = 0.553).

Supplementary Figure 1


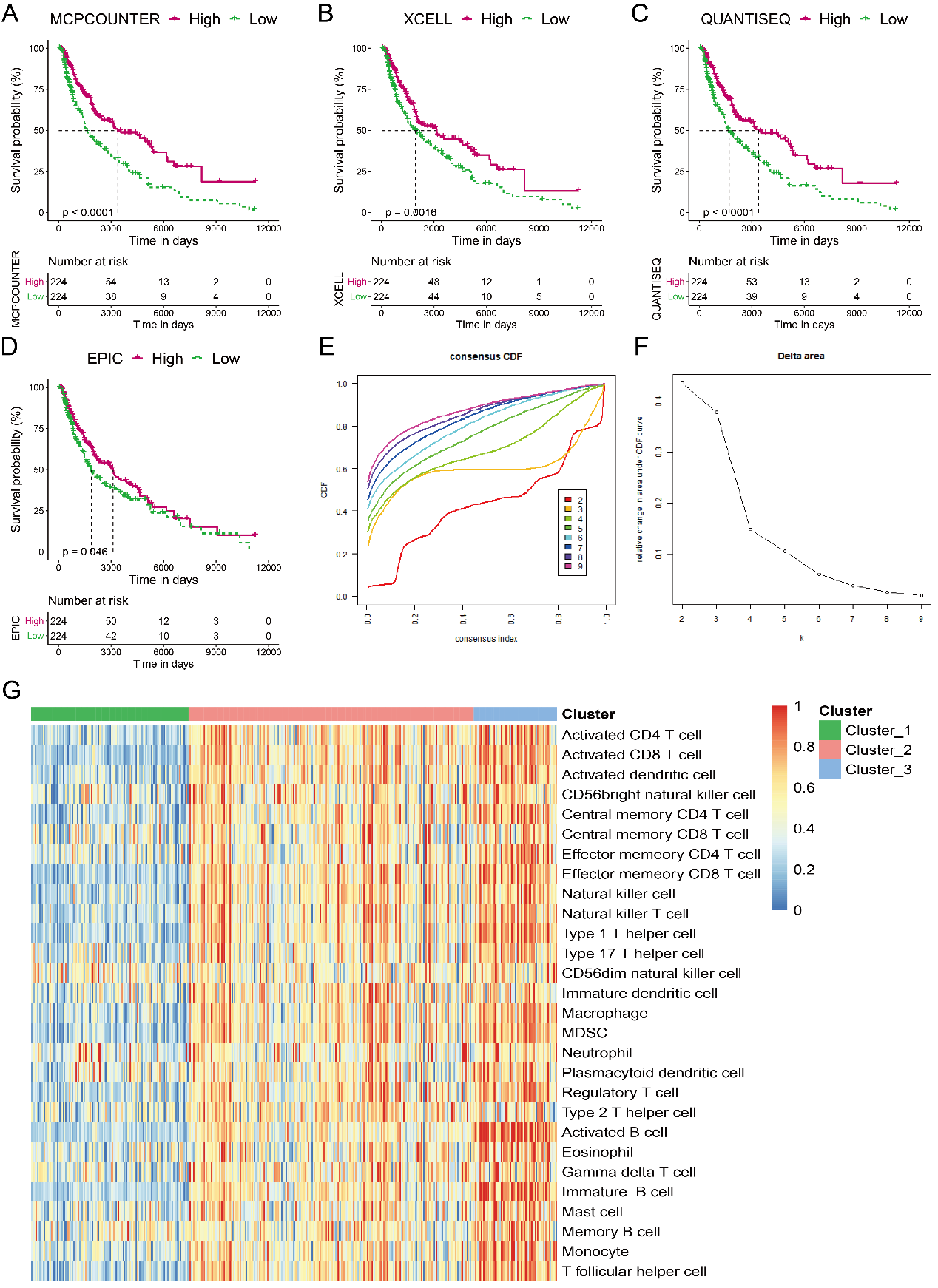


Supplementary Figure 2


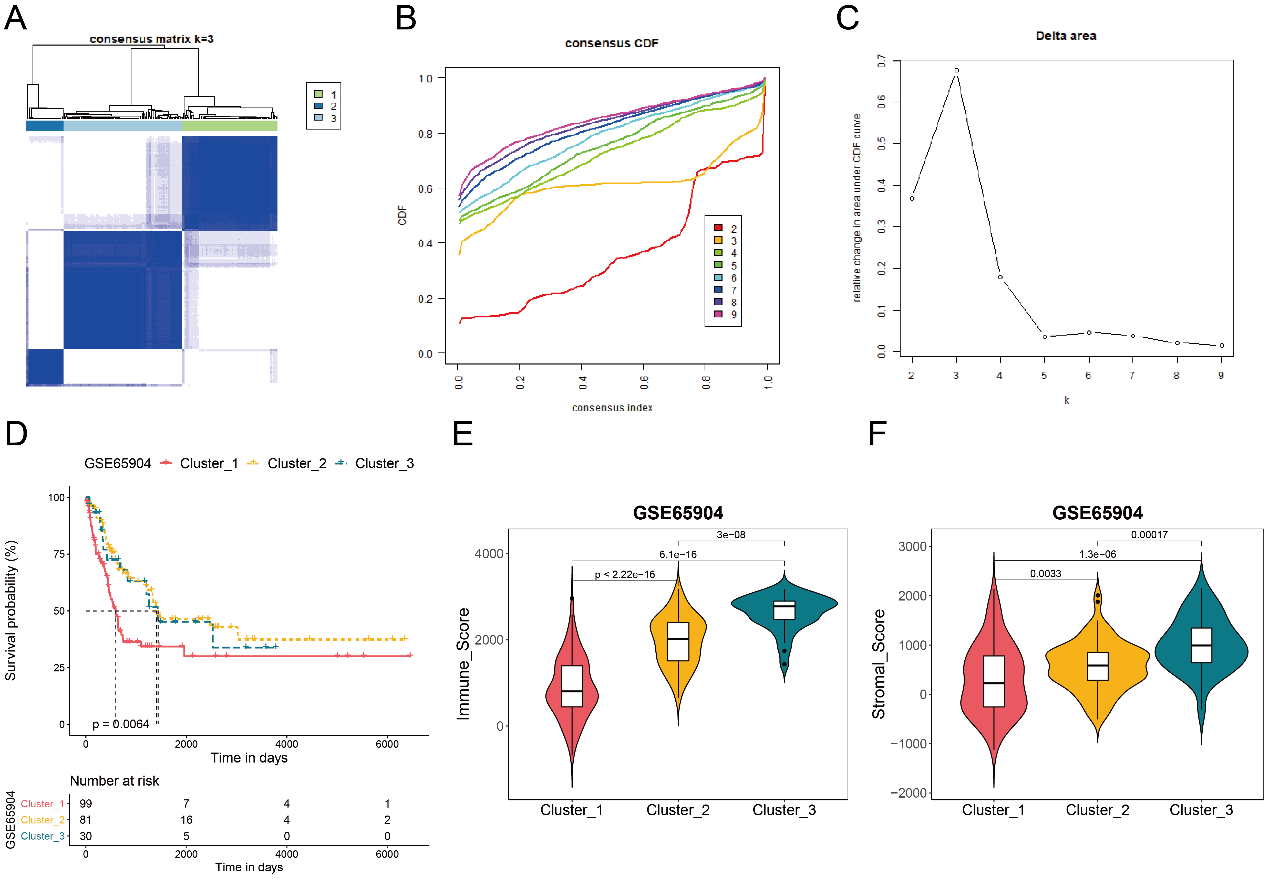


Supplementary Figure 3


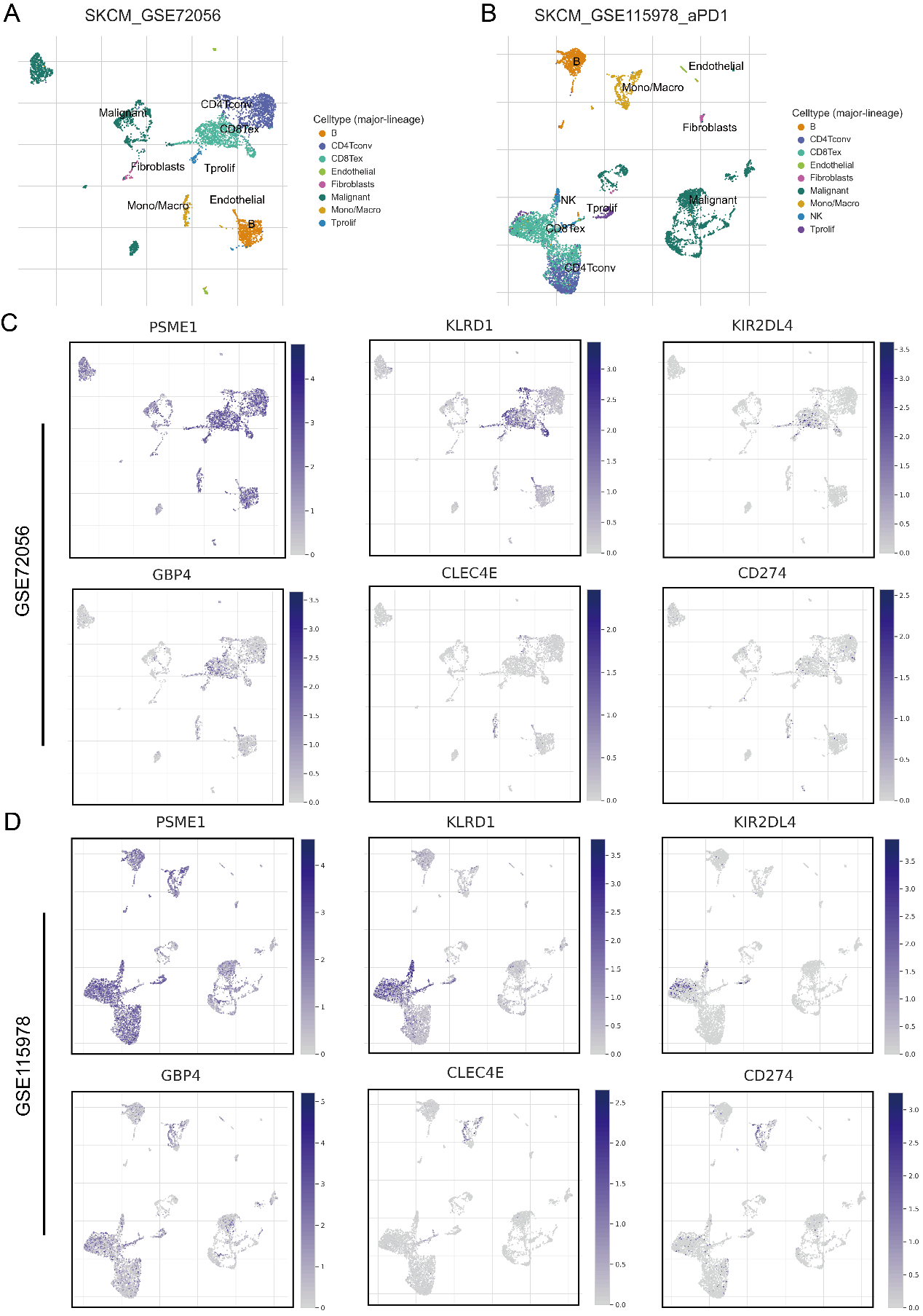


Supplementary Figure 4

**
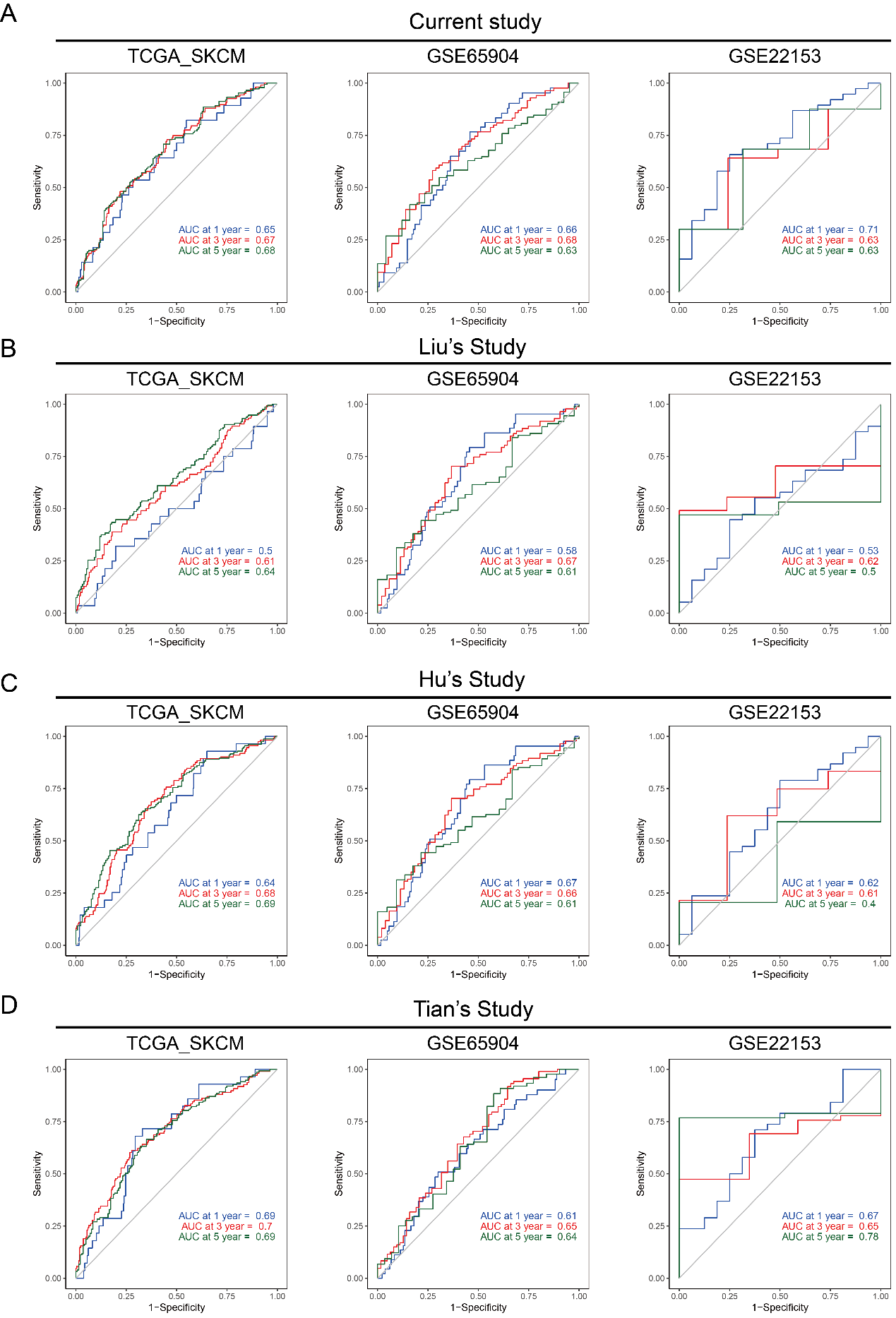
**

Supplementary Figure 5

**
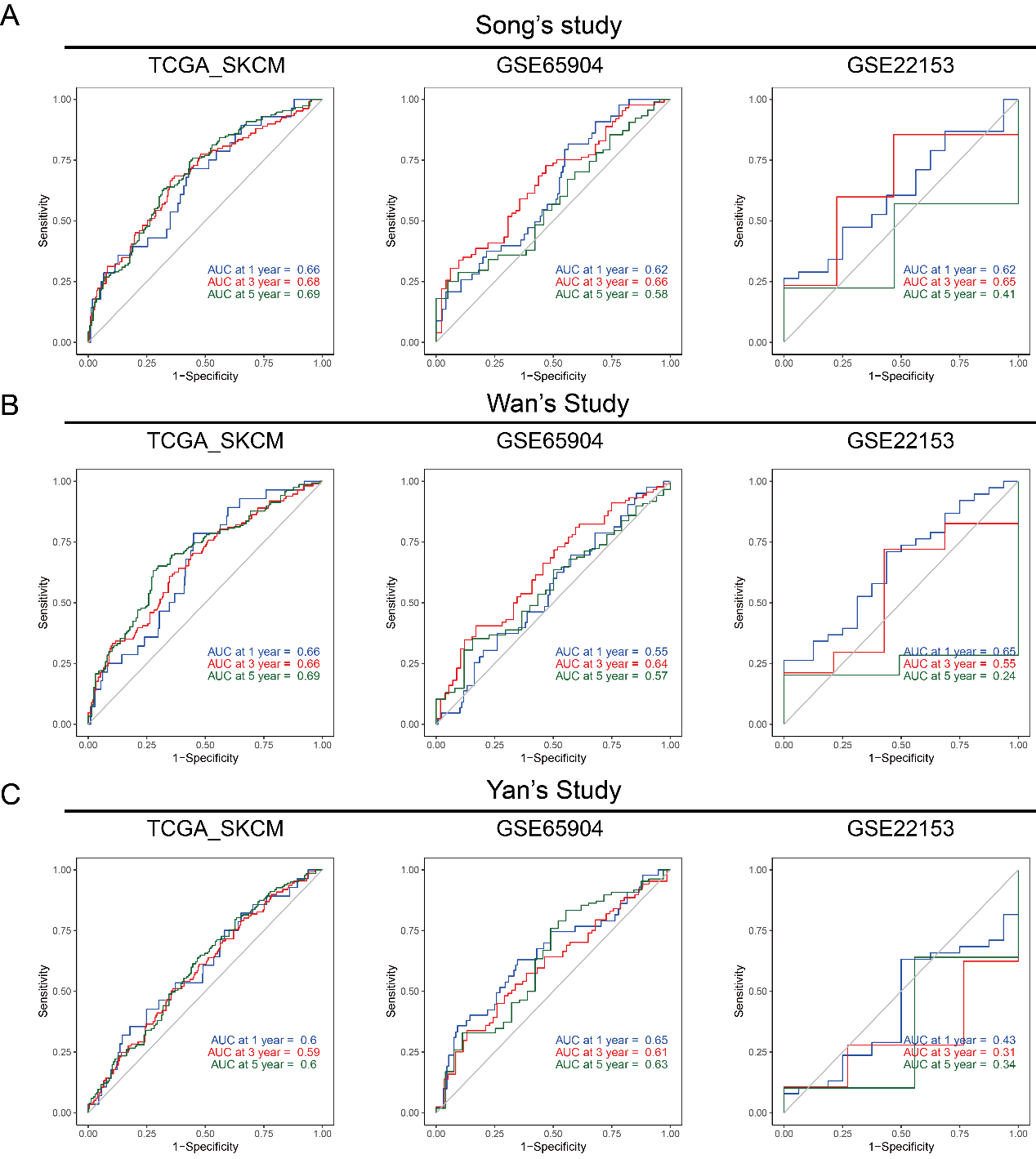
**

Supplementary Figure 6

**
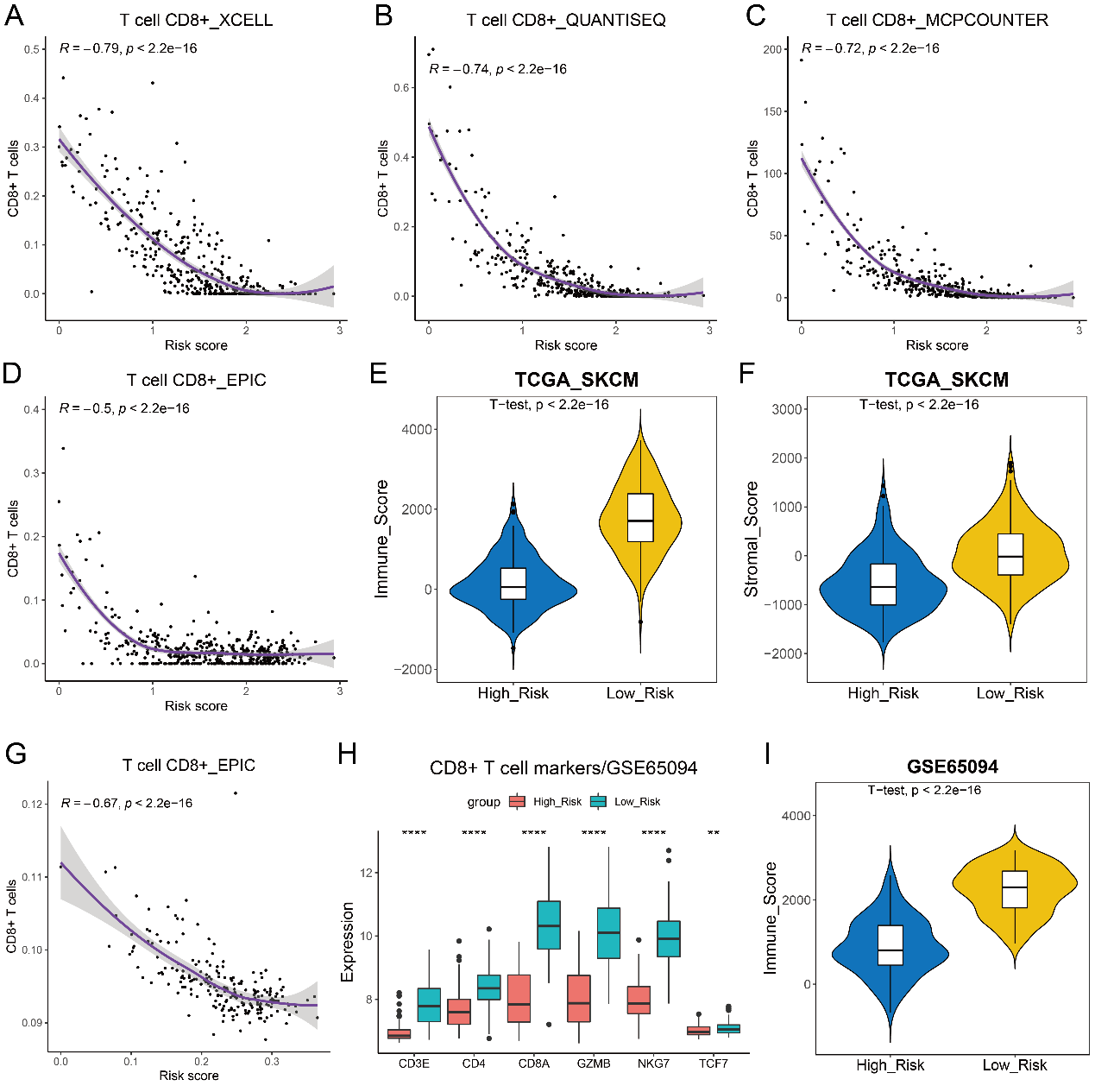
**

Supplementary Figure 7

**
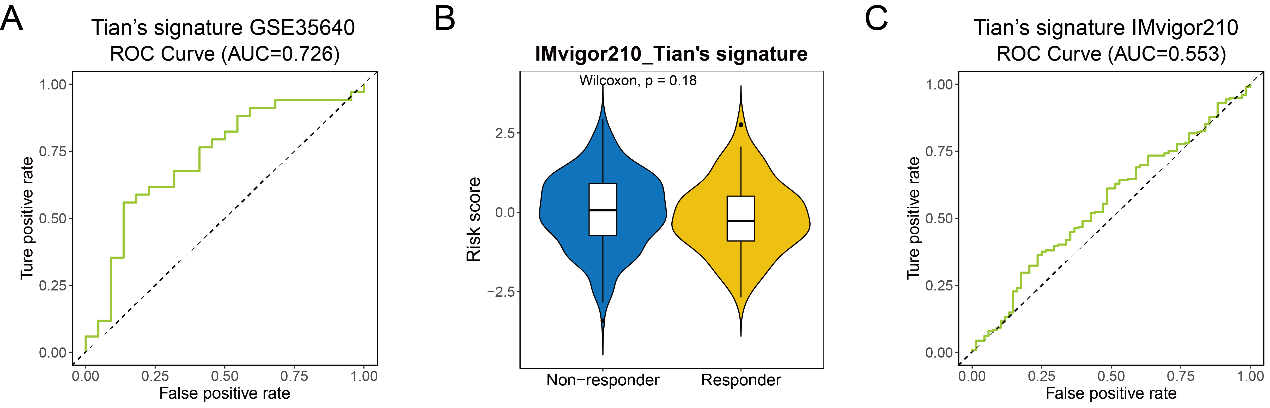
**
